# *“The One Who Chases You Away Does Not Tell You Go”*: Silent Refusals and Complex Power Relations in Research Consent Processes in Coastal Kenya

**DOI:** 10.1371/journal.pone.0126671

**Published:** 2015-05-15

**Authors:** Dorcas M. Kamuya, Sally J. Theobald, Vicki Marsh, Michael Parker, Wenzel P. Geissler, Sassy C. Molyneux

**Affiliations:** 1 KEMRI-Wellcome Trust research Programme, Kilifi, Kenya; 2 The Ethox Centre, Nuffield Department of Population Health, University of Oxford, Oxford, United Kingdom; 3 Centre for Tropical Medicine, Nuffield Department of Medicine, University of Oxford, Oxford, United Kingdom; 4 Department of International Public Health, Liverpool School of Tropical Medicine, Liverpool, United Kingdom; 5 Department of Social Anthropology, Oslo University, Oslo, Norway; 6 Department of Social Anthropology, University of Cambridge, Cambridge, United Kingdom; London School of Hygiene and Tropical Medicine, UNITED KINGDOM

## Abstract

Consent processes have attracted significant research attention over the last decade, including in the global south. Although relevant studies suggest consent is a complex negotiated process involving multiple actors, most guidelines assume consent is a one-off encounter with a clear ‘yes’ or ‘no’ decision. In this paper we explore the concept of ‘silent refusals’, a situation where it is not clear whether potential participants want to join studies or those in studies want to withdraw from research, as they were not actively saying no. We draw on participant observation, in-depth interviews and group discussions conducted with a range of stakeholders in two large community based studies conducted by the KEMRI Wellcome Trust programme in coastal Kenya. We identified three broad inter-related rationales for silent refusals: 1) a strategy to avoid conflicts and safeguard relations within households, - for young women in particular—to appear to conform to the wishes of elders; 2) an approach to maintain friendly, appreciative and reciprocal relationships with fieldworkers, and the broader research programme; and 3) an effort to retain study benefits, either for individuals, whole households or wider communities. That refusals and underlying rationales were silent posed multiple dilemmas for fieldworkers, who are increasingly recognised to play a key interface role between researchers and communities in many settings. Silent refusals reflect and reinforce complex power relations embedded in decisions about research participation, with important implications for consent processes and broader research ethics practice. Fieldworkers need support to reflect upon and respond to the ethically charged environment they work in.

## Background

Consent for participation in health research in low income countries is imbued with many widely documented complexities. These include difficulties in explaining and understanding research terminologies [1–3], participants joining research primarily to access much needed health care [2, 4–6], and involvement and influence of family and other community members in individual decision-making [7, 8]. Although consent remains a core theme in research ethics, the extent to which entirely autonomous individual informed consent is possible, or even desirable, is an area of on-going ethical debate particularly in highly communal and patriarchal communities [9, 10].

Numerous guidelines and documents describe strategies to strengthen consent processes in an effort to meet key ethical principles of respect to persons and communities. A common recommendation is that consent be seen as a process [11, 12], and that research involves several layers of communication and consultation with host communities in addition to individual consent processes [13, 14]. The relationship between community engagement (CE) and individual consent processes is far from straightforward. For example, while community engagement can facilitate improved knowledge of a study in the host population [14–16], too much information has the potential to lead to key research information being crowded out [13, 17], and for people to feel obliged to participate in research in order for the community to gain benefits provided in research [18].

In some situations, involving a potential participant’s partner or other relative (‘significant other’) in decision-making is recommended [1, 19–21]. However there is relatively little research on the nature of consultations between individuals and their significant others, the influence of such consultations on decisions, or the ethical importance of such influences in low income settings. These are important areas to explore since the nature of negotiations between research staff, potential participants, and their significant others can vary considerably from simple information giving to interactions where clear power asymmetries and potential for coercion exist. A particular research gap concerns decision-making in research ‘decliners’, not least because this information is practically and ethically challenging to collect [22, 23].

Within these complex and negotiated processes, the staff who undertake informed consent, often called frontline research staff or fieldworkers (FWs), play a key role. It is widely recognised that employing fieldworkers from study communities is essential for many research studies [24, 25]. Beyond conducting consent processes in the first language of potential participants, FWs take on many other important roles—formally or informally-including: conducting relatively straightforward research procedures such as simple surveys and health checks [26, 27]; providing access to hard-to-reach populations such as stigmatized or hidden populations [28]; advising researchers on local priorities, concerns, and culturally appropriate conduct of research including consent [29] and acting as ‘cultural brokers’ between the often very different worlds of researchers and research communities. A growing body of literature about such frontline workers suggests that their roles are imbued with diverse and ever-changing ethical challenges with important implications for consent processes [30–33]. For example, some frontline staff may be motivated by their wish to help their communities [24, 27], with potentially positive and negative implications for consent processes, including excessively encouraging research participation in contexts of constrained public health care systems [33]. Where fieldworkers are already well known within communities, there is potential for them to exploit this trust to meet recruitment quotas, and for confidentiality to be compromised [26, 27]. Given their central role in ethical consent processes, there is increasing recognition of the need to appropriately support fieldworkers in their community interactions; and for research guidelines and ethics frameworks to build on their experiences.

Many documents aimed at strengthening consent processes include a core assumption that potential participants will—at some point in the process—explicitly agree or refuse to join a proposed study. Those who agree can then later change their minds [34]. However, a clearly stated decision may not be a reality. For example, in a previous study in Kenya, community members were argued to exert agency during interactions with relatively well-resourced researchers and institutions through a number of strategies that included ‘silent refusals’; that is, hesitating to participate without explicitly refusing [31, 35]. In this paper we present one theme- ‘silent refusal’—that emerged from a broader social science study which explored the nature of interactions between fieldworkers and participants in two community-based studies in coastal Kenya. We describe the nature of silent refusals as it emerged in the community based studies, how fieldworkers encountered the silent refusal, the challenges and dilemmas they faced in handling silent refusals. We further make some recommendations for consent processes and for support to fieldworkers.

### Study Site: KEMRI-Wellcome Trust Research Programme

The study was carried out in a long-standing international research centre, the KEMRI-Wellcome Trust Research Programme (KWTRP); established on the Kenyan Coast since 1989 and with branches in other parts of the country including Nairobi (http://www.kemri-wellcome.org/). At the Coast, KWTRP is hosted at the Kilifi County Hospital (KCH), with research and support for Ministry of Health (MOH) services conducted in tandem. The centre runs a Health and Demographic Surveillance System (KHDSS) including about 260,000 residents living around KCH, representing the geographic area in which most studies are conducted in Kilifi [36].

All studies conducted by the Programme are approved by the national scientific and ethics review committees in addition to institutional—and where necessary external—review committees [37]. A comprehensive community engagement includes programme-wide and study specific activities [38]. A group of staff, the Community Liaison Group (CLG), consisting of experienced community facilitators, coordinates centre-wide community engagement activities, and advises all studies on community engagement at every stage of a study. Issues arising from the community are fed back to relevant departments and study Principal Investigators (PIs).

Fieldworkers are the largest group of staff at the research centre, forming nearly a third of the staff [31]. They are often recruited from the community where a study is conducted. Their main roles include communicating about studies, undertaking consent processes and following-up participants at their homes.

## Methods

**Fig 1:**
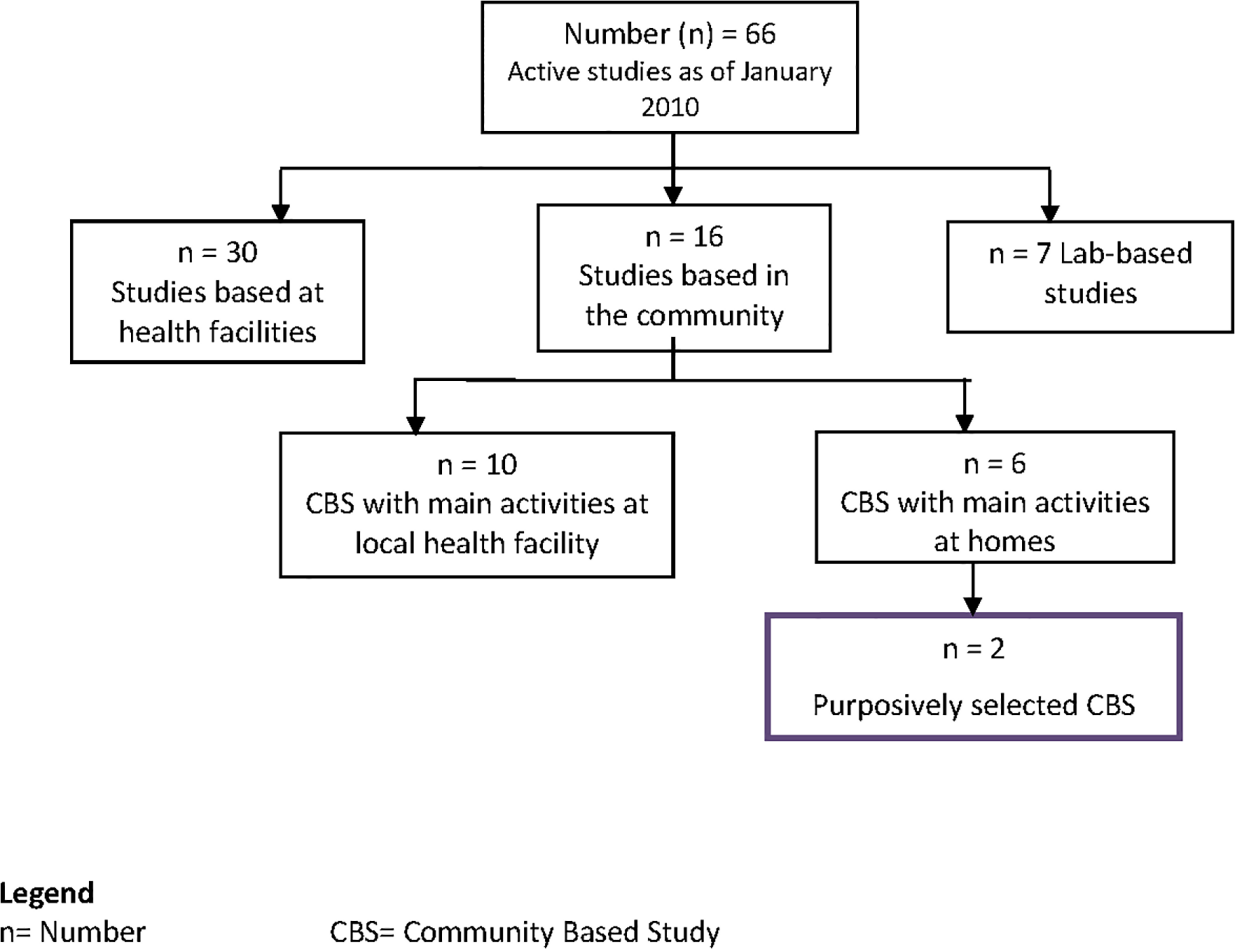
Flow-Chart showing selection of the community-based studies.

The data presented in this paper were collected as part of a wider social science study aimed at exploring the nature of interactions between fieldworkers and research participants in community-based studies, the challenges that fieldworkers faced, and if and how these were resolved. We purposively selected two community based studies for this broader social science study using criteria presented in Table 1. **Fig 1**shows the flowchart of the selections of the two studies from a total of 66 active studies at the research centre at the time, of which 16 were community-based studies. The two community-based studies selected were:

- An observational basic science study involving entire households (n = 47 households) examining respiratory syncytial virus transmission patterns (RSV-study, within KHDSS);
- A malaria vaccine trial involving 900 children from Kilifi divided into two groups, 6–12 weeks and 5–17 months (Malaria-study; outside KHDSS). The vaccine trial was a multi-site study involving eleven sites in seven African countries; this qualitative research focused on the Kilifi site.

**Table 1:** Criteria considered in selecting the Community–based studies.

Criteria	Justification
Number of fieldworkers	Selected studies with more than one FW to be able to cross check information across different respondents, minimise responder bias—the tendency to respond desirably and explore FWs dynamics in a team
Where FWs were based	Studies with community-based FWs selected to
	enable exploration of how social embeddedness of FWs might influence research participation
	Participants would easily understand who FWs are, as opposed to ward-based FWs who may be mistaken for doctors
Amount of interaction with participants	Selected on-going studies with considerable FW-participant interaction to minimise recall bias, and to learn nature of those interactions
	studies involving different amounts of interactions i.e. entire household, vs an individual participants
	studies at different stages of the research cycle as likely to affect nature of interactions
Sensitivity of procedure or information and levels of benefits	sensitivities around study procedures likely to impact on amount of time taken, range of issues FWs encountered, and nature of relationship between FWs and the participants
	selected studies with different study procedures which are considered sensitive (e.g. included blood/nasal swabbing/vaccine)

The key features of the two community-based studies are published elsewhere [39] and summarised in Table 2, including the main research questions each for each study, type of participants, locality, study team composition, study benefits and risks.

**Table 2:** Key features of the two community based case studies.

Feature	RSV-study	Malaria-study
Study question/ objective	Who is infecting whom in the household with Respiratory Synctial Virus (RSV)	Evaluate the efficacy of a ‘promising’ malaria candidate vaccine against malaria disease in infants and children, and across diverse malaria transmission settings in Africa.
		The study aimed to address key safety and efficacy information required for vaccine licensure
Study design	Basic science descriptive study.	Double blind (observer blind), randomized, controlled, multi-centre study.
Study period	Oct 2009–April 2010; participant involvement for 6 months.	2008–2015, participants’ involvement for 34 months.
Number of study sites	One site in KEMRI-WT, Kilifi.	Eleven sites in seven countries; Burkina Faso, Gabon, Ghana, Kenya, Malawi, Mozambique and Tanzania.
Study area in Kilifi	One location, 15 kms from the Kilifi District Hospital (KCH), within KHDSS.	Three administrative divisions, 30 kms from KCH, 5 locations, in Kilifi County.
Composition of study team	16 team members which consisted shared staff *of lab technicians, drivers; 10 ‘Junior’ FWs, 2 data entry clerks, one each SFW, clinician, coordinator, PI and senior researcher.	47 staff which included shared staff* with other projects of data entry clerks, lab technicians, drivers; 26 ‘Junior’ FWs, 5 SFWs, 3 clinicians, 2 Medical officers, and one each of study coordinator, PI and senior researcher.
Participants	Entire household in a defined locality with an infant born after previous RSV epidemic and at least one elder sibling to the infant. Household defined as all members of a homestead who share at least one meal a day.	Children aged 6–12 weeks and 5–17 months at first vaccination; 16,000 children across the 11 sites, a minimum of 6,000 in each of the age category. *For Kilifi site*, *allocated total of 900 children*, *600 and 300 in the 5–17 months 6–12 weeks group respectively*.
Study procedures	Follow-up visits at home every 3–4 days; data from each HH member collected at each visit included:	Randomisation to one of three groups; Experimental malaria vaccine and its booster at 1.5 years
	Temperature measurement, a nasopharyngeal flocked swab (NFS); history of respiratory illness	Experimental malaria vaccine and a different booster dose of either Meningitis and septicaemia vaccine; and
	In addition, respiratory rate taken for all children under 5 years.	Three doses of rabies vaccine plus a different booster doses of Meningitis and septicaemia.
	Flocked oral swab (taken at alternate visits (once a week).A demographic and risk assessment questionnaire administered at beginning and end of the study.	***Procedures*:** Initial physical examination, medical history, anthropometric tests, temperature; three vaccine doses each a month apart, and booster dose at 34 months; 5 scheduled blood samples over 3 years; each 2.5mls; Monitoring of minor and serious adverse; immediate and over time; 6 consecutive follow-up visits post-vaccination days at home. Monitoring of minor and serious adverse events; Referral to nearest health facility for illnesses, and to KCH for serious illnesses.
Risks	Mild discomfort during NSF taking, and time inconveniences.	Detailed side effects as is typical of vaccines trials provided in the study protocol and informed consent; includes severe (such as convulsions, diarrhoea) and mild events (e.g. pain, swelling at vaccination site).
Benefits	**For *participants*:** Free medical care for all common illnesses during study period; clinical visits to every participating household once a month at home.	***For participants***: Free health care for all conditions (chronic, acute, vaccine related or otherwise, injuries), throughout the study period (about 3 years). This includes:
	Other benefits/token staggered throughout the study period included two chairs to each household, sweets, educational materials and token** at end of study.	Free referral for specialized treatment where required, all costs at government facilities covered while transport is provided for first visit to non-government facilities.
		All transport to and from the hospital provided by the study team and meals provided for participant and accompanying parents/guardian for al clinic visits.
	***Community benefits***: boosting local health services through provision of drugs, additional clinical staff. Water treatment for all communal water points	***Community benefits***: Boosting of three health facilities where the study is based; renovation of existing buildings, providing equipment’s; boosting of health staff, provision of essential drugs.
	Provision of emergency medical aid during cholera epidemic including drugs, staff, referrals.	

*These staff were shared with other studies within the department

**Token given at the end of the study were said to be the study teams appreciation to participants for having persevered until the end of the study. They included educational materials, food items, clothes to family members

A total of 36 ‘Junior’ FWs and 6 Senior FWs were employed across these case studies. All ‘Junior’ fieldworkers came from and resided within the study population, and most were male (7/10 and 25/26 in the RSV- and Malaria-study respectively). Their main roles included sharing initial study information with potential participants and carrying out follow-up activities.

The social scientists carrying out this study were independent of the case study research teams. DK had over 8 years of managing and coordinating community engagement activities, training and providing support to fieldworkers at the research centre, with support from SM and VM. This knowledge, experience and relationships were important in building trust and being aware of different perceptions informing the findings. The main data collection methods were participant observation, natural and focus group discussions and individual in-depth interviews. All the immediate study team members in the two community based studies, that is, ‘Junior’ fieldworkers (n = 36), senior fieldworkers (n = 6), study Principal Investigators (n = 2) and study coordinator (n = 1) and were interviewed. In addition, we interviewed purposively selected participants in each case study. Table 3 shows the demographic characteristic of the respondents interviewed in this social science study.

**Table 3:** Demographic characteristics of respondents.

Socio-demographic characteristic	RSV-study (Number)	Malaria-study (Number)
**Fieldworkers (FWs)**		
Total number	10	26
Gender (female)	3/10	1/26
Mean age, years (range)	26.5 (20–34)	27.81 (21–38)
<24 years	4	7
25–29	3	10
30–34	3	6
35–39	9	12
Marital status (married)	2	9
Education, average (range) years of schooling	12.2 (12–14)	12.31 (12–14)
12 years—O-level-	9	22
14 years—College/diploma	1	4
Average period (months) worked at KWTRP	7.3 (5–9)	10.13 (0.1–16)
< = 5 months	4	5
6–10 months,	6	9
11–15 months,	None	4
16–20 months,	None	8
Number of FWs with relatives participating in the study	1	11*
Contract period offered*	9 months	2 years
**Senior Fieldworkers**		
Total number	1	5
Gender (males, %)	1 (100%)	5 (100%)
Age in years (mean, range)	36 years	29.2 (25–35)
24–29yrs, (number, %)	N/A	3 (60)
30–34yrs	N/A	2 (20)
35–39yrs	1 (100)	2 (20)
Employment duration (mean, range)	12 years	4.8 (3–8)
1–5yrs, (number, %)	N/A	3 (60)
6–10yrs	N/A	2 (40)
11–15yrs	1	N/A
**Respondent-participants in the two Community-Based studies**		
Number of respondent for social science study	16	29
Gender (female)	11/16 (69%)	15/29 (52%)
Age in years (mean, range)	26.2 (20–40)	34.9 (18–63)
<24 years (number, %)	4 (25)	4 (14)
25–34	10(63)	12 (45)
35–44	2 (13)	9 (31)
45–54		2 (7)
>55		2 (7)
Education levels (Number, %)		
None	3 (19)	9 (31)
1–4 years	6 (38)	7 (24)
5–8 years	7 (44)	12 (41)
9–12 years		1 (3)

*as at the time of collecting the data in early 2010.

### Participant observation

Participant observation provided first-hand information of the context in which FWs worked and the type and nature of interactions between FWs and different householders. DK carried out participant observations for a total of 4 months in the RSV-study and 1 month in the Malaria-study, and attended 12 and 4 study meetings respectively. She visited 19 households in the RSV-study and 30 participant households in the Malaria-study; and accompanied all 10 RSV ‘Junior’ FWs and 9/26 Malaria-Study ‘Junior’ FWs during their daily work. Considerable time was spent the first study selected for the social science study—in the RSV-study- to get deeper understanding of the range and depth of issues in FW-participant interactions. Less time was spent in the Malaria-study as the aim was to explore the extent to which findings from RSV-study were generalisable to a different type of community-based study in the same context.

### Group discussions and in-depth interviews

A total of 11 focus group discussions (FGDs) with 64 respondents, 5 natural group discussions with 16 respondents and 7 in-depth interviews with 4 respondents were held (Table 4). Natural group discussions were held with adult household members all participating in case study A, as this approach was one way of exploring household decision-making dynamics [40]. The advantage of natural group discussions is that members already know one another, and have established some norms of working as a group, which the research can gather insights from. Because some of the topics we explored in these natural groups turned out to be sensitive (such as household decision-making dynamics), we used FGDs in subsequent interviews.

**Table 4:** Summary of interview methods and respondents.

Interview type	Respondents
In-depth interviews (IDI)	6 IDIs with RSV-study researchers, two each with PI, Study coordinator, Senior FW
	1 IDI with one FW in the Malaria-study
Natural group discussions	5 Natural (household) group discussions with 16 adults participating in the RSV-study
Focus Group Discussions (FGD)	3 FGDs with 10 FWs in the RSV-study (one group interviewed twice)
	3 FGDs with 26 Field workers in the Malaria-study
	4 FGDs with 24 participants (grouped per gender) in the Malaria-study
	1 FGD with 5 SFW in the Malaria-study

Respondent-households in the RSV-study were selected from the 19 households DK had previously visited during her participant observation. In this way, DK was not a total stranger and had some idea of the household dynamics. Within this group, households were purposively selected to reflect diversity based on gender and household arrangements (extended and nuclear families). Respondent-households in the Malaria-study were purposively selected from the geographical area surrounding each of the three health facilities in which the trial was being conducted. FGDs were held separately with male and female respondents to address sensitivities around gender roles and household decision-making for research, as noted above. In both case studies, discussions were held with the study team members separately; that is fieldworkers and senior fieldworkers, study coordinator and principal investigators.

Written informed consent was sought from interviewees in their preferred language. One RSV household refused to participate and did not want to disclose the reason.

### Data management and analysis

Data collection continued until a point of saturation where no new themes were emerging. Data analysis started as soon as the first interviews were transcribed and cleaned, and continued throughout the study. All cleaned transcripts were uploaded into Nvivo Version 8.0, the software we used to organize and manage the data. Immediately after each step of data collection, DK and SM printed and read the transcripts, identified emerging issues from each transcript, and DK made summaries. We chose the most informative fieldworker FGD for initial open coding in Nvivo Version 8.0, as it would provide the most variable themes and categories [41]. Data under each open code were grouped into descriptive themes, and codes were merged, deleted and created as more transcripts were added [42]. Through this iterative process of analysis further areas of enquiry were identified and incorporated into subsequent question guides. The descriptive codes were further grouped into broader analytical themes. DK and SM independently coded initial transcripts and compared these with those of another independent researcher. The framework so developed was discussed with ST, PWG, VM and MP. To safeguard participants’ privacy and confidentiality, all individual identifiers were replaced with codes in transcripts and in write ups. Summary findings were presented to different cadres of staff in the case studies and to researchers at the centre, as part of validating the findings.

### Ethics Statement

This study was approved by the local and national Institutional review Boards (IRB); the KEMRI Scientific Steering (SSC) committee and the KEMRI Ethics Review Committee (ERC), SCC protocol number 1463.

## Findings

### Setting the scene for silent refusals: How were consent processes negotiated in the two case studies?

**Fig 2:**
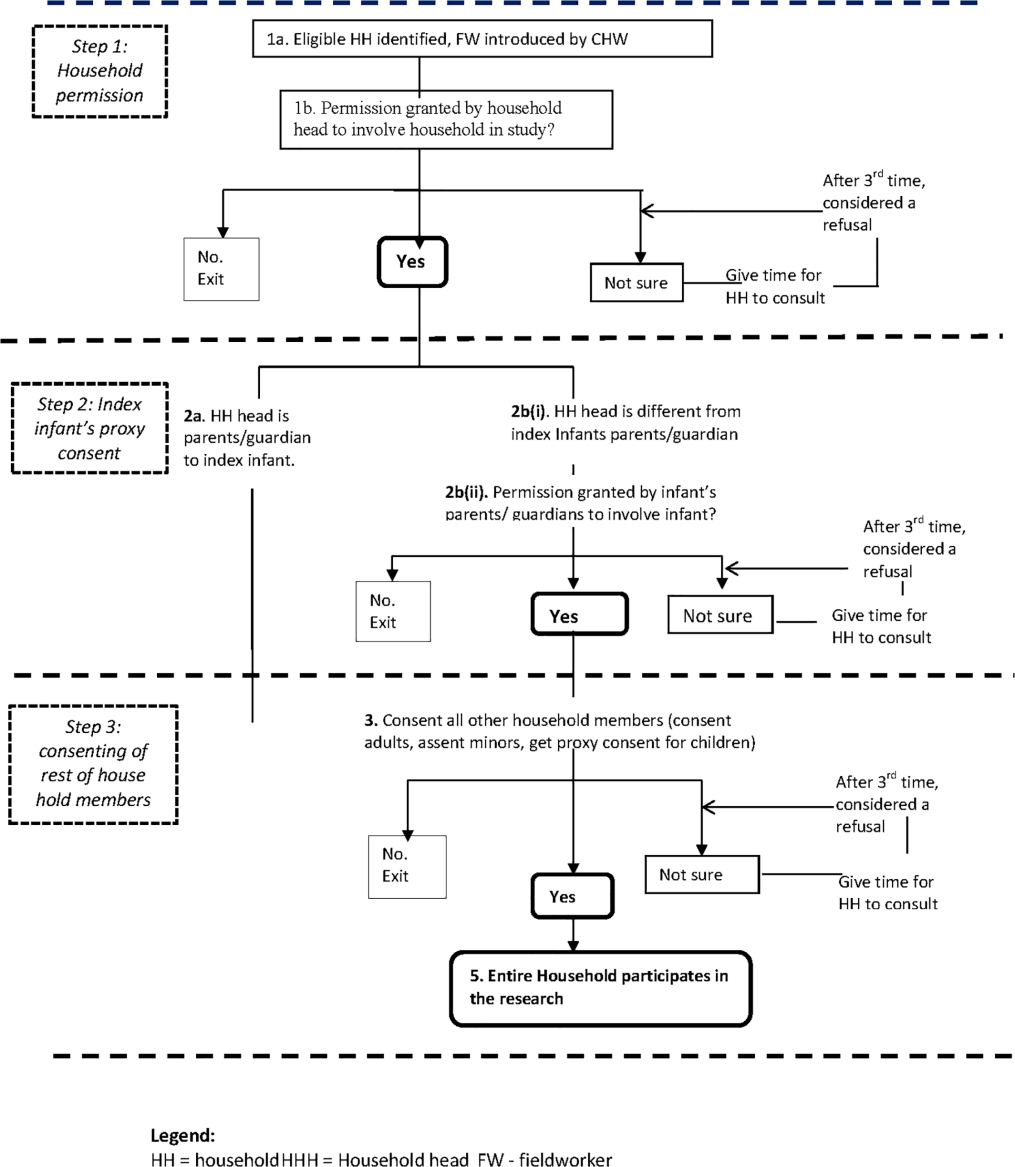
Consenting process for RSV-study.

**Fig 3:**
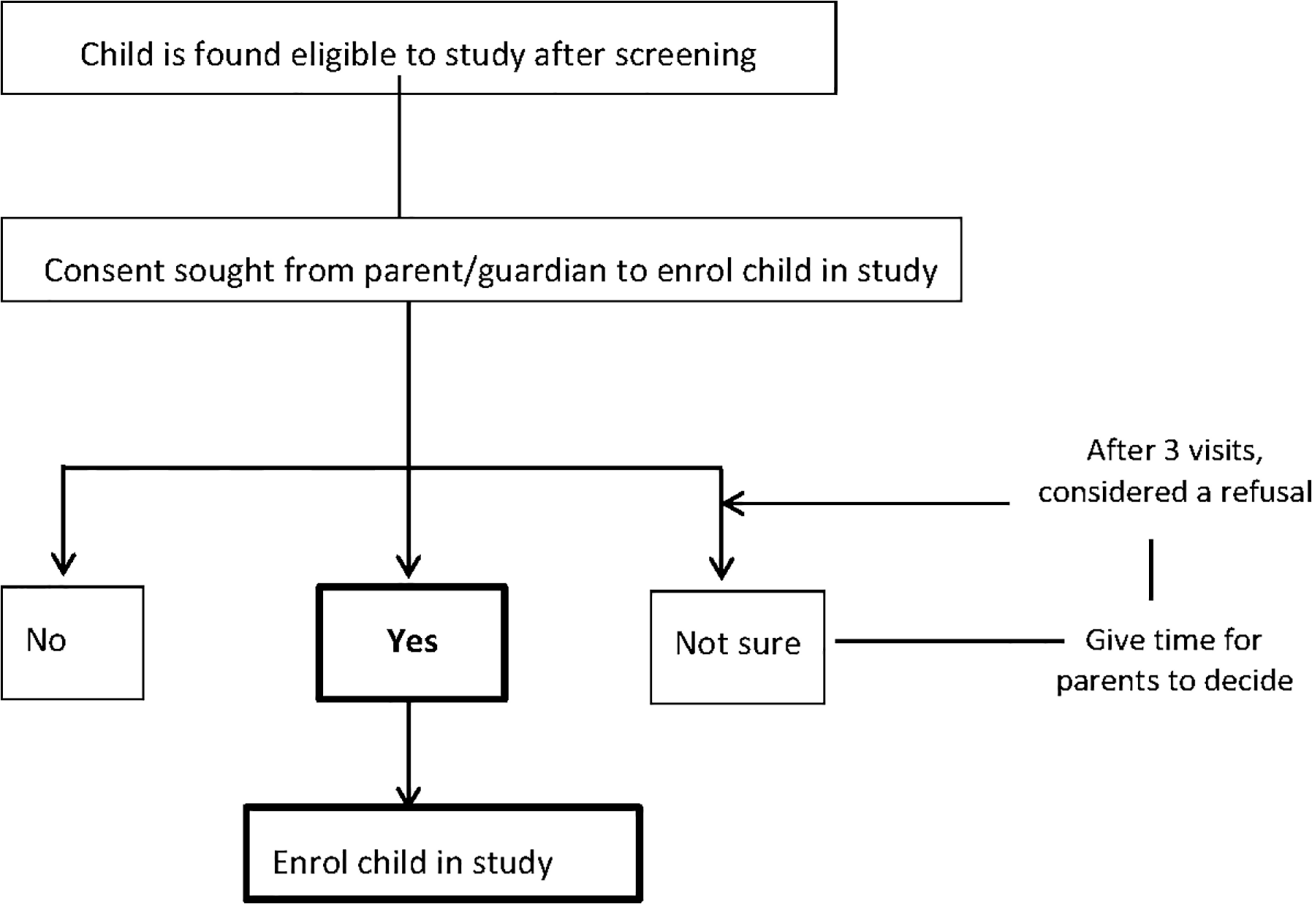
Consenting process for Malaria-Study.

Individual and household consent processes in the case studies changed over time in response to practical challenges the teams encountered. For example, the RSV study initially sought consent from all adults in a household and assent from minors, but over time, the importance of gaining initial permission specifically from male household heads was recognised (Fig 2). For the Malaria study, the community facilitators in the CLG initially shared information about the trial to groups of potential participants as part of Community engagement activities for that trial. This was followed by parental consent by a clinician at the health facility (Fig 3). Over time and once the FWs had been trained in the trial details the FWs took the role of explaining the trial to potential participant.

Study benefits provided in case studies (See Table 2) were highly appreciated constantly discussed and negotiated, and ultimately contributed centrally to whether participants joined and remained in studies. Particularly appreciated were the high quality and prompt health care services given, and the respectful way research staff handled participants. Food and transport for participants and guardians during follow-up visits (Malaria-study) and provision of chairs, educational materials, and first aid to the whole community during a cholera epidemic (RSV-study), were seen as responding appropriately to community needs. However, such provisions also seemed to contribute to expectations of ever-increasing benefits, and potential jealousies between participants and non-participants.

Concerns and worries which fed into continuous negotiations in the two studies included the safety of Nasopharyngeal Flocked Swabs (NFS) which were taken twice a week from all household members for 6 months (RSV-study), the safety of the malaria vaccine, concerns over blood samples (Malaria-study), and about the nature and motivation of the work of KWTRP.

Across community engagement activities, consent, daily research activities and study completion, DK observed constant discussions around benefits, and of participants’ concerns and worries of being involved in the studies. A particular challenge for FWs was how to handle (potential) participants who repeatedly postponed consent decisions or who participated inconsistently. These were often referred to as ‘silent refusals’ by study teams and across the research programme.

### Silent refusals: What are they and how did they emerge?

Silent refusals were observed during consent processes and over the course of studies. During consent processes, ‘silent refusal’ was used to describe the behaviour of those who appeared positive about participation, but kept postponing their final decision, often with very ‘good’ reasons. For example, some mothers requested extended periods to consult their husbands, mothers-in-law, or other family members, even when these individuals had already agreed to research participation. The need to involve others was expected and encouraged, and to avoid putting undue pressure on individuals, FWs were trained to follow up to a maximum of 3 times before categorising this response as a refusal. However FWs found it difficult to adhere to this guidance where individuals really appeared interested to participate, but did not make a commitment. Post-consent, a ‘silent refusal’ was used to describe those who participated in some but not all study procedures, but who appeared reluctant to withdraw from studies. They often had credible reasons for their inconsistent participation that did not suggest a desire to withdraw:

“… but the way they were telling you is that, even you (FW) go in the morning and you tell them (participants) ‘the vehicle is coming, prepare yourself’…and she tells you ‘there is no problem’. … and you go there with the vehicle, and when you arrive there she says ‘aaah I have remembered I have a visitor, so I will not come to the dispensary’” (*FW1_male_FGD10*).

Initially FWs found it difficult to discern a silent refusal, but over time, as they got to know individuals and households better, this pattern became easier to identify. As one FW pointed out using a popular Kiswahili proverb:

“…I have truly known that *akufukuzae hakwambii toka* [the one who chases you away does not tell you go]… you have to understand them according to their actions or words then you completely know that this person does not want to participate” (*FW1_male_FGD05*).

**Why silently refuse?** The reasons for silent refusals appeared to fall under three broad inter-related categories: avoiding conflicts in the household and with significant others; safeguarding relationships between participants and FWs/KWTRP; and ensuring continued access to study benefits.

#### Avoiding conflicts and safeguarding relations within households

A silent refusal was described as a norm when politely refusing people who were strangers, or who are highly respected. An outright refusal is perceived as being rude. Being polite is seen as a good in itself, and as an investment in kindness and generosity for future situations of need, even from strangers.

“… so you don’t want to refuse someone’s request because it’s like you are *unaweka akiba* [investing] so that another day someone will be kind to you” (*FW3_male_FGD06*).

Within households, showing respect—especially to household heads and elders—is highly valued. Children and women should show this through obeying. A silent refusal is a way to disagree with elders and household heads respectfully, without openly disobeying them. With regards to research consent, decisions about household and children’s participation in research were often made by male household members, even where consultations did take place. Those most affected by participation, often women charged with taking children for follow up procedures, wanted to be actively involved in making decisions about joining studies. Where this did not happen, silent refusals appeared to be a subtle strategy to covertly exercise agency. As a mother narrated, women would often only cooperate in studies involving their children if they supported the decision or would use different tactics to avoid research participation.

“…the mother is the one who will get hold of the child as blood is being drawn. If the father agrees for the child to participate but the mother refuses then you (mother) won’t send the child. You may go and stay outside and then go back (home) with your child and say the child has missed the vaccines or you say the child has been attended; but you will just be cheating. … you will hold the child during bleeding, you look at her as she cries, but you will only do so if you have decided…” (*Pax2_female_FGD12*).

In these instances, silent refusal was used by women as a short term strategy to mask a genuine refusal to participate in ways that did not cause rifts within households. The same strategy was used by younger men towards older male household members or heads.

“… So the thing is if like that one (a silent refusal), he could not out rightly say that he had withdrawn because he is not the household head, his elder brother [the household head] fully participates in the study…” (*FW4_male_FGD06*).

It seemed that male participants were aware that their wives (and minors) used silent refusals to delay, and sometimes influence, their decisions; but felt unable to do anything about it. Instead they seemed to blame women and minors for not understanding the importance of the health care ‘assistance’ provided by KWTRP.

“Yes, as the husband you can consent, and the wife can go as if going to the dispensary and because of ignorance (not knowing) you can be saying the child has been sent (to the dispensary)… but in fact he has not been enrolled in the study. And if she comes to the dispensary, she does other things. So because of that they (study researchers) keep waiting for her and they get tired…” (*Pax6_male_FGD13*)

An additional concern was reported widespread jealousy of husbands toward male FWs, given the latters’ relatively well-paid jobs at KWTRP. Some men felt uneasy with male FWs visiting their wives at home, especially in the evening. Similarly, some female participants described being careful in associating with male FWs given these potential conflicts, instead choosing to participate in studies when their husbands were away—in order to access study benefits—and refusing at other times.

#### Safeguarding relationships with FWs and with KWTRP

Similar views to those within household relations were raised with regards to research, KWTRP and FWs. Participants knew that health care provided in KWTRP studies was prompt and of ‘high quality’ and wanted to continue accessing it. Some participants also perceived KWTRP to have great influence on the general provision of health care in public health facilities, despite reassurances to the contrary. As a well-resourced powerful research institution in a relatively poor community, some participants found it difficult to decline research participation given concerns about offending individual FWs and KWTRP, and used silent refusals to respectfully avoid participation because of fears that lack of cooperation would affect access to health care:

“…one knows KEMRI has a branch here (at the dispensary). So they fear that ‘if I say I don’t want KEMRI here…if my child or I become sick and I go there (at the dispensary) and I had refused KEMRI (research), then I may be denied services’” (*FW2_male_FGD10*).

Silent refusals were also described as a strategy to delay decision-making in situations where participants were unsure of the implications of their choices, or where they wanted to further investigate the credibility of study information given; for example, by waiting to see if participants developed side effects. They worried that openly requesting for additional time to make decisions might be considered impolite or as a refusal:

“You see, silent refusal usually happened because someone sees like you have become a member of that family; you have become part of them. So, they start to blame themselves because you have that *uhusiano* (good relationship) between you and her. So she feels if she tells you openly that…‘from now or from today I withdraw’…she feels guilty that ‘this person has been like my child, has been like my grandchild, or has been like my uncle or has been like a brother, so when now I withdraw its like I will be chasing them away… So that is why you see them not showing the signs of wanting to withdraw” (*FW1_male_FGD06*).

For FWs who come from the community where the studies were conducted, positive relationships with participants were consolidated over the course of the studies, with FWs becoming considered part of families in many households [31]. Being employed in KWTRP gave FWs recognition and high status in the community. In addition there may have been an expectation for mutual reciprocity between FWs and participants; that is, that FWs would help community members through enrolling them in studies and ensuring access to study benefits; in turn the community members would participate in research. Therefore, participants may not have wanted to offend FWs by declining participation, instead using silent refusals tactfully.

“Somebody like me, I’m respected in the community. So, somebody coming to me and telling me that I don’t want that (study) is hard. So he takes you round, like the saying ‘the one who chases you doesn’t tell you go away’, so its upon you to understand that… because she respects, she can’t tell you no” (*FW5_male_FGD10*).

Silent refusals may also have been a strategy to avoid participation in studies participants had not intended to join initially but had done so under perceived pressure from peers, friends and community leaders. Rumours about KEMRI generally, and research in particular—especially blood sampling—may have contributed to this form of silent refusal.

#### Study-related concerns: not wanting to lose study benefits

Study benefits formed one of the most common reasons for joining and remaining in studies, as we have described in a separate paper [39]. Fear of losing study benefits may have contributed to reluctance to refuse or withdraw from studies. In this case, silent refusals seemed to be used to mask active participation within households, as a form of ‘silent participation’, when some household members wanted to access or continue accessing benefits in the absence of permission from recognised decision-makers. In this case, they ‘covertly’ consented to participate when key dissenting adults were absent. They hoped that over time, the dissenters’ fears would ease, allowing them to participate openly. The extent to which FWs were aware of this, and might even have encouraged it, was difficult to know. That it happened, albeit in few cases, is a point of concern for consent processes.

“Maybe the vehicle goes to pick her…when the father is around, he refuses the mother from going to the dispensary. But when the husband is not around… she comes to the dispensary” (*Pax9_male_FGD13*).

FWs were aware that study benefits filled a livelihood need especially for the extremely poor households; allowing silent refusals to remain in the study was one way of helping such families. The flipside for FWs was that accommodating silent refusals was likely to encourage other participants to behave similarly. This could potentially affect the quality of the research data collected.

### Handling silent refusals

#### Study team handling of silent refusals.

Protocols in both case studies stated that participants should be followed up to a maximum of three times, after which he/she is considered a refusal and dropped from the study. The Malaria-study protocol also stated that follow-up visits are made to withdrawers to know the reasons, to provide important information should the vaccine eventually be licensed. Consent forms clearly stated that participation is voluntary and that a participant can withdraw at any time without censure, and would continue receiving health care services normally. Each study also required a minimum number of participants to be scientifically valid, allowing for dropouts and withdrawals.

When faced with silent refusals, FWs and researchers initially followed study protocols and dropped participants from the study. However, they worried that high levels of refusals would affect the eventual validity of the study, particularly for the RSV-study where NFS was unfamiliar and majority of the participants initially declined to have it taken. FWs responded by spending extra time with participants to reassure them, giving opportunities for extended discussion to address concerns and clarify information. This contributed to generally positive relations between FWs and participants, and to building of trust as we describe elsewhere [31]. The positive relationships developed between participants and FWs, and the mutual benefits of working with silent refusals—as described above—safeguarded against hasty decisions. In the RSV-study, over time the team accepted some silent refusals as ‘permanent’ within a participating household if an individual repeatedly ‘not available’ had minimal contact with an index child.

“…we accommodated those who rarely get in to contact with the infant…whenever we did our home visits, we would collect data on their illness history…I would say they were not actually refusals of study participation, it’s refusals to be swabbed [taken the NFS] period; but they were happy to be in the study…”, (R1_male_IDI04).

#### Participants’ views on how study teams should handle silent refusals

Both men and women participants in the case studies suggested that FWs should find out underlying reasons for silent refusals and find ways to resolve them. This included providing more information and addressing misunderstandings about the study and KWTRP’s work. Only one participant suggested that those reluctant to participate in research should be withdrawn from the study.

“…every time I fail to get the mother at home [because she dodges], then I will want to know the reason, whether she is willing to participate or not” (Pax9_ female_FGD12).

The strategies suggested for addressing silent refusals differed between men and women participants, related to the gendered nature of household decision-making in this community [43]. Many women felt that community leaders such as village elders and chiefs should arbitrate between FWs and silent refusals, men saw such arbitration as challenging their decision-making authority:

“In their misunderstanding they think if they go to the village elder they have been sued [challenged] for a mistake they have done. Instead of them knowing that they are being assisted with their children getting treatment they think they are going to be arrested. So these advices we are getting here, if he is called by the village elder he will think he’s been sued…,” (*Pax1_male_FGD15*).

Those suggesting arbitration appeared to base it on wanting to support participants’ access to high quality free health care, arguably a misunderstanding on what research is and how it differs with treatment; and the place for personal choice in research.

### FWs’ dilemmas in handling silent refusals

FWs and researchers saw an importance in understanding the reasons behind silent refusals in order to address these. Some FWs felt frustrated when they encountered silent refusals, being confused as to whether repeated follow-up visits would be a nuisance, and concerned about the increased workload. The genuine nature of reasons given made it difficult to strictly adhere to the rule of a maximum of three follow-up visits. In addition, FWs worried that failing to give participants enough time to make decisions could lead to tensions between themselves and participants. In a few instances, FWs felt that their Principal Investigator (PIs) did not understand these dynamics well enough, instead tending to blame FWs for causing a rift with participants, leading to silent refusals.

“… so that is the problem for us (FWs) …it’s better if you didn’t consent the person in the study but … she is in the study … she has gotten one dose (of vaccine); its better if that person had relocated to another area, there would be no problem. But you know she is there (at home) and she doesn’t want to tell you (why she is reluctant) and then when you reach here (office) there is pressure you are being asked, ‘what have you done to this child? What have you done to the mother until she now wants to withdraw from the study?’” (F*W1_male_FGD10*).

Participants were aware that silent refusal posed particular challenges for FWs, and that the complex nature of underlying reasons and the fact it is not an openly expressed choice made it difficult for FWs to know how to response appropriately [39]. The dilemma for FWs with silent refusals was whether to accept it as a refusal and drop participants from the study or retain them. Dropping participants risked annoying some, adversely impacting relationships between them and the FW [31] and creating difficulties where FWs were continuing to visit other participants in the same and neighbouring household. Dropping participants also meant they could no longer access study benefits, an issue FWs struggled with especially for the poorest households [39]. In addition, over time participants appeared to get used to ‘unpopular’ study procedures, so that a FW deciding to drop a participant might later be blamed by that participant for taking such action, as narrated by a FW.

….you see silent refusal usually happened because someone sees like you have become a member of that family…so he/she feels if they tell you openly from now or from today I withdraw there may be a problem…because the family will not qualify to continue to be in the study, (*FW1_male_FGD6*)

Finally, as FWs live and are embedded within communities beyond the study period, they were understandably careful that transient research activities should not spoil already established long-term relationships [31]. With regards to the science of the study, by retaining silent refusals FWs feared they might collect low quality data, requiring repeated explanation to PIs. Whichever course of action FWs took, there were likely to be strained relationships, even if temporarily, between the FWs, participants and researchers.

## Discussion

This study showed the socially embedded processes of consent processes for community-based studies in a long-term well-resourced research centre operating in a relatively poor, largely patriarchal community on the Kenyan Coast. In particular, we have described situations in which research consent decisions are not stated with an outright yes or no response. The findings highlight that negotiations were taking place throughout the course of the research; within households, between participants and FWs, and with others. While consent is one of the most widely described areas in research ethics, there is surprisingly little detailed research on the nature of these negotiations, and on how negotiations occur in research consent decisions. This paper further highlights that while ethical principles such as respect for persons and autonomy maybe universal, the way these are interpreted and negotiated can be context-specific, with implications for how individual agency is exercised. Given the paucity of literature on silent refusals in research conduct, there is need for empirical research in this area and across different research types and settings.

In focusing on research decision negotiations, and on silent refusals as a complex set of strategies within those negotiations, this study has illustrated that consent decisions are made in the lived social world of participants; with decisions not only about weighing up research importance, risks and benefits, but also about safeguarding important relations with significant others and researchers. In this context silent refusals emerged as a strategy used to, among others, maintain harmonious relationships within household and with FWs, to negotiate participants’ favourable participation levels while accessing full study benefits. In other papers, we have described the centrality of social relations [31], study benefits [39] and of gender roles and household dynamics (in preparation) in shaping research participation in community-bases studies in our setting. In this paper we focus on silent refusals in the context of complex and unequal power relations during research conduct.

Silent refusals highlight the interweaving of power relationships into research processes. They highlight agency and the ways in which normative power relations can be challenged and overturned; wives making decisions about areas they would otherwise not be expected to control; and participants, to some extent, determining how to participate in research. VeneKlasen and Miller, cited in [44], describe power as “…both dynamic and multi-dimensional, changing according to context, circumstances and interests. Its’ expressions can range from domination and resistance to collaboration and transformation” (p3). Long’s description of agency power appears to be reflected in the concept of silent refusal.

“[Power] is the outcome of complex struggles and negotiation over authority, status, reputations and resources … such struggles are founded upon [the] extent to which specific actors perceived themselves capable of manoeuvring within particular situations and developing strategies for doing so” [45]p2.

Thus power can be exerted both overtly such as directly influencing actions and choices of others, and/or covertly such as subtle strategies that can influence the dynamics of negotiations [46]. In this study, silent refusals illustrate complex power relations within households, and between FW and households.

### Within household power relations

Authoritative power, or power over others, [44] in households was largely described as held by male household heads over other household members, from elders to younger members, and from older women (such as first wives) to younger female (and male) household members. This form of power appeared to work in practice in some households, and on certain occasions, but not in others. Thus, some female household members and minors appeared to challenge male dominance in situations where they felt their choices mattered, particularly where research decisions and participation was likely to affect their roles in the household. Kandiyoti’s (1988) concept of bargaining with patriarchy appropriately frames some of the strategies women and minors used to resist male dominance in some aspects of the research [47]. Because of the covert nature of silent refusal (unexpressed refusal), those with authoritative power such as husbands, household heads and male household members expressed helplessness when they encountered it. The phenomenon of silent refusal suggests that, at least in the Kilifi setting, participants’ choices about research participation are influenced by the way the research is likely to shape relationships with significant others in the household. It was used to circumvent unpopular research decisions, and to manoeuvre personal preferences into decision-making processes, while also maintaining harmony in key relationships. Strategies for negotiating favourable decisions in unequal power relations within households have been documented in household treatment seeking behaviours for seriously ill children ([43]; however there relatively little information with regards to research participation.

### Participant-FW (and study) relations

With regards to power in participant-FW relations, generally it seemed that FWs were respected in the community due to their being employed in a reputable organisation (KWTRP) and having access to resources, technical knowledge, information about the study and access to the PIs. Participants thus conceptualised FWs as community gatekeepers and expected them to put community interests over and above those of KWTRP and of research. FWs were likely to be aware of these expectations, and may have encouraged them to achieve their own goals such as attaining high recognition and meeting recruitment targets, as documented elsewhere [48]. FWs were also aware that entering people’s homes put them in positions of vulnerability, since household members had control over if and how the FWs would be received. FWs thus used discretionary power [44] during research implementation to shape research conduct, and to influence perceptions of community members and PIs about them, and about the study. Thus, overall, one would expect FWs to have power over participants in their interactions.

Participants in the case studies were aware that it was important for them to follow study procedures consistently for the research to be successful. This was discussed in various forums including in community engagement meetings, during consent processes and at follow-up visits. They also knew that FWs’ job performance might largely depend on their participating faithfully in the research. It, therefore, seems that participants had latent power [46] to influence research conduct, through their ability to determine whether the study would take place (and in what form), and whether FWs would be welcomed to their homes for follow-up visits. They exercised these powers in various ways; overtly through continuous discussions; and covertly particularly through silent refusals. Together, these factors contributed to participants’ perceptions and understanding of their tacit power to control the direction of study implementation, while maintaining harmony in key relationships. We call this subtle power as it was not explicitly expressed and appeared scattered and fragmented across different participants and over time. The relational nature of this form of power made it difficult to detect in the first instance, and was frustrating to those who experienced it, especially the FWs. The practice of silent refusal showed that to some extent participants could exert agency in their participation levels (choosing what study procedures to participate in, when and how), and the overall research implementation. Exploring factors underpinning silent refusals shows an intricate interplay between multiple sources of power amongst participants and FWs that shaped decisions about research participation.

Many of the findings serve to highlight the disconnect between universalist ethics approaches and individual/contextualised responses to medical research. This tension is constantly faced by FWs in their daily roles, as something they have to constantly negotiate although it is rarely acknowledged. There is now a growing body of literature that is drawing attention to the ethical challenges and dilemmas that fieldworkers face in undertaking their research roles in developing countries[25, 26, 32, 49]; this paper adds to this body of literature by describing an area that has not been written about, silent refusals and subtle power negotiations in research participation.

## Conclusion

While many communities exhibit some form of silent refusal, very little has been written about it with regards to research participation. Silent refusals illustrate the complexities inherent in negotiating decisions around research participation and the ongoing nature of informed consent processes. They illuminate the socially embedded nature of research participation, and the significance of taking account of the social worlds of participants and the ways in which agency is exercised to shape research implementation in otherwise unequal power relations. Exercising such agency calls us to take account of both individualism and the relational nature of those involved in research, and the sometimes conflicting ways these two play out; such as women subtly exercising their power to make decisions in areas they otherwise would not be involved in, but doing so in ways that avoid conflicts within and beyond the household. Recognising the centrality of negotiations for research participation is one way to take account of power relations embedded in such processes, and to start to unpack and understand the ethical issues around consent as a process. In this research, we support the call to consider consent as a process for community-based studies. We propose that this requires understanding the nature of consultations and negotiations that go on, and unpacking of ethical issues interwoven in such processes. Those involved in undertaking consent, often fieldworkers who are embedded in communities, play a critical interface role and mediate between researchers and the community; one which brings multiple dilemmas and challenges. The complexity of power relationships between participants and FWs is echoed in those between FWs and PIs. PIs need to be aware of the ethically charged, complex and fluid environments that FWs encounter in their daily work. Guidelines are unlikely to be adequate for all situations that FWs encounter, and PIs who are more removed from the messy realities of community-embedded consent processes will find it difficult to understand the issues. Providing constructive supportive supervision where there is ‘space’ for fieldworkers to air and discuss their own embedded knowledge and concerns—as much as possible throughout the entire research cycle—will be important, as will ensuring that FWs are trained in research ethics, communication skills, and how to recognise and respond to ethical dilemmas they may encounter.

## References

[1] Molyneux CS, Peshu N, Marsh K. Understanding of informed consent in a low-income setting: three case studies from the Kenyan Coast. Social science & medicine. 2004;59(12):2547–59. Epub 2004/10/12. 10.1016/j.socscimed.2004.03.037 PubMed . 15474208

[2] Fairhead J, Leach M, Small M. Where techno-science meets poverty: medical research and the economy of blood in The Gambia, West Africa. Social science & medicine. 2006;63(4):1109–20. Epub 2006/04/25. 10.1016/j.socscimed.2006.02.018 PubMed . 16630676

[3] Benatar SR. Reflections and recommendations on research ethics in developing countries. Social science & medicine. 2002;54(7):1131–41. Epub 2002/05/10. PubMed . 11999507 10.1016/s0277-9536(01)00327-6

[4] Lidz CW, Appelbaum PS. The therapeutic misconception: problems and solutions. Medical care. 2002;40(9 Suppl):V55–63. Epub 2002/09/13. 10.1097/01.MLR.0000023956.25813.18 PubMed . 12226586

[5] Masiye F, Kass N, Hyder A, Ndebele P, Mfutso-Bengo J. Why mothers choose to enrol their children in malaria clinical studies and the involvement of relatives in decision making: evidence from Malawi. Malawi medical journal: the journal of Medical Association of Malawi. 2008;20(2):50–6. Epub 2009/06/23. PubMed 19537433 10.4314/mmj.v20i2.10957 PMC2748955

[6] Mfutso-Bengo J, Ndebele P, Jumbe V, Mkunthi M, Masiye F, Molyneux S, et al Why do individuals agree to enrol in clinical trials? A qualitative study of health research participation in Blantyre, Malawi. Malawi medical journal: the journal of Medical Association of Malawi. 2008;20(2):37–41. Epub 2009/06/23. PubMed 19537430 10.4314/mmj.v20i2.10898 PMC3345665

[7] Ndebele P, Mfutso-Bengo J, Masiye F. HIV/AIDS reduces the relevance of the principle of individual medical confidentiality among the Bantu people of Southern Africa. Theoretical medicine and bioethics. 2008;29(5):331–40. Epub 2008/12/03. 10.1007/s11017-008-9084-y PubMed . 19048391

[8] Molyneux CS, Peshu N, Marsh K. Trust and informed consent: insights from community members on the Kenyan coast. Social science & medicine. 2005;61(7):1463–73. Epub 2005/07/12. 10.1016/j.socscimed.2004.11.073 PubMed . 16005781

[9] Irabor DO, Omonzejele P. Local attitudes, moral obligation, customary obedience and other cultural practices: their influence on the process of gaining informed consent for surgery in a tertiary institution in a developing country. Developing world bioethics. 2009;9(1):34–42. Epub 2009/03/24. 10.1111/j.1471-8847.2007.00198.x PubMed . 19302568

[10] Ho A. Relational autonomy or undue pressure? Family's role in medical decision-making. Scandinavian journal of caring sciences. 2008;22(1):128–35. Epub 2008/02/14. 10.1111/j.1471-6712.2007.00561.x PubMed . 18269432

[11] Tekola F, Bull SJ, Farsides B, Newport MJ, Adeyemo A, Rotimi CN, et al Tailoring consent to context: designing an appropriate consent process for a biomedical study in a low income setting. PLoS neglected tropical diseases. 2009;3(7):e482 Epub 2009/07/22. 10.1371/journal.pntd.0000482 PubMed 19621067 PMC2705797

[12] Participants in the Community E, Consent Workshop KKM. Consent and Community Engagement in diverse research contexts. J Empir Res Hum Res Ethics. 2013;8(4):1–18. Epub 2013/10/31. 10.1525/jer.2013.8.4.1 PubMed . 24169417 PMC4836561

[13] Marsh VM, Kamuya DK, Parker MJ, Molyneux CS. Working with Concepts: The Role of Community in International Collaborative Biomedical Research. Public health ethics. 2011;4(1):26–39. Epub 2011/03/19. 10.1093/phe/phr007 PubMed 21416064 PMC3058176

[14] Rotimi C, Leppert M, Matsuda I, Zeng C, Zhang H, Adebamowo C, et al Community engagement and informed consent in the International HapMap project. Community genetics. 2007;10(3):186–98. Epub 2007/06/19. 10.1159/000101761 PubMed . 17575464

[15] Doumbo OK. Global voices of science. It takes a village: medical research and ethics in Mali. Science. 2005;307(5710):679–81. Epub 2005/02/05. 10.1126/science.1109773 PubMed . 15692036

[16] Lavery JV, Tinadana PO, Scott TW, Harrington LC, Ramsey JM, Ytuarte-Nunez C, et al Towards a framework for community engagement in global health research. Trends in parasitology. 2010;26(6):279–83. Epub 2010/03/20. 10.1016/j.pt.2010.02.009 PubMed . 20299285

[17] Marsh VM, Kamuya DM, Mlamba AM, Williams TN, Molyneux SS. Experiences with community engagement and informed consent in a genetic cohort study of severe childhood diseases in Kenya. BMC medical ethics. 2010;11:13 Epub 2010/07/17. 10.1186/1472-6939-11-13 PubMed 20633282 PMC2918624

[18] Molyneux S, Mulupi S, Mbaabu L, Marsh V. Benefits and payments for research participants: experiences and views from a research centre on the Kenyan coast. BMC medical ethics. 2012;13:13 Epub 2012/06/26. 10.1186/1472-6939-13-13 PubMed 22726531 PMC3407030

[19] Reynolds L, Cousins T, Newell M-L, Imrie J. The social dynamics of consent and refusal in HIV surveillance in rural South Africa. Social Science & Medicine. 2013;77(0):118–25. 10.1016/j.socscimed.2012.11.015. 23219165 PMC3560061

[20] Beskow LM, Botkin JR, Daly M, Juengst ET, Lehmann LS, Merz JF, et al Ethical issues in identifying and recruiting participants for familial genetic research. American journal of medical genetics Part A. 2004;130A(4):424–31. Epub 2004/09/30. 10.1002/ajmg.a.30234 PubMed . 15455364

[21] Marshall PA, Adebamowo CA, Adeyemo AA, Ogundiran TO, Strenski T, Zhou J, et al Voluntary participation and comprehension of informed consent in a genetic epidemiological study of breast cancer in Nigeria. BMC medical ethics. 2014;15:38 Epub 2014/06/03. 10.1186/1472-6939-15-38 PubMed 24885380 PMC4032563

[22] van Bogaert LJ. Rights of and duties to non-consenting patients—informed refusal in the developing world. Developing world bioethics. 2006;6(1):13–22. Epub 2006/01/27. DEWB132 [pii] 10.1111/j.1471-8847.2006.00132.x PubMed . 16436170

[23] Williams B, Irvine L, McGinnis AR, McMurdo ME, Crombie IK. When "no" might not quite mean "no"; the importance of informed and meaningful non-consent: results from a survey of individuals refusing participation in a health-related research project. BMC health services research. 2007;7:59 Epub 2007/04/28. 10.1186/1472-6963-7-59 PubMed 17462081 PMC1866231

[24] Mosavel M, Ahmed R, Daniels D, Simon C. Community researchers conducting health disparities research: Ethical and other insights from fieldwork journaling. Social science & medicine. 2011;73(1):145–52. Epub 2011/06/18. 10.1016/j.socscimed.2011.04.029 PubMed 21680071 PMC3126882

[25] Molyneux S, Kamuya D, Madiega PA, Chantler T, Angwenyi V, Geissler PW. Field workers at the interface. Developing world bioethics. 2013;13(1):ii–iv. Epub 2013/03/26. 10.1111/dewb.12027 PubMed 23521824 PMC3662993

[26] Simon C, Mosavel M. Community members as recruiters of human subjects: ethical considerations. The American journal of bioethics: AJOB. 2010;10(3):3–11. Epub 2010/03/17. 10.1080/15265160903585578 PubMed 20229402 PMC3139466

[27] Madiega PA, Jones G, Prince RJ, Geissler PW. 'She's my sister-in-law, my visitor, my friend'—challenges of staff identity in home follow-up in an HIV trial in Western Kenya. Developing world bioethics. 2013;13(1):21–9. Epub 2013/03/26. 10.1111/dewb.12019 PubMed 23521821 PMC3674534

[28] McKnight C, Des Jarlais D, Bramson H, Tower L, Abdul-Quader AS, Nemeth C, et al Respondent-driven sampling in a study of drug users in New York City: notes from the field. Journal of urban health: bulletin of the New York Academy of Medicine. 2006;83(6 Suppl):i54–9. Epub 2006/09/16. 10.1007/s11524-006-9102-1 PubMed 16977493 PMC1705505

[29] Kamuya D, Marsh V, Molyneux S. What we learned about voluntariness and consent: incorporating "background situations" and understanding into analyses. The American journal of bioethics: AJOB. 2011;11(8):31–3. Epub 2011/08/03. 10.1080/15265161.2011.583328 PubMed . 21806436

[30] Kingori P. Experiencing everyday ethics in context: frontline data collectors perspectives and practices of bioethics. Social science & medicine. 2013;98:361–70. Epub 2013/11/12. 10.1016/j.socscimed.2013.10.013 PubMed 24210881 PMC3898703

[31] Kamuya DM, Theobald SJ, Munywoki PK, Koech D, Geissler WP, Molyneux SC. Evolving friendships and shifting ethical dilemmas: fieldworkers' experiences in a short term community based study in Kenya. Developing world bioethics. 2013;13(1):1–9. Epub 2013/02/26. 10.1111/dewb.12009 PubMed 23433316 PMC3662996

[32] Chantler T, Otewa F, Onyango P, Okoth B, Odhiambo F, Parker M, et al Ethical challenges that arise at the community interface of health research: village reporters' experiences in Western Kenya. Developing world bioethics. 2013;13(1):30–7. Epub 2013/03/26. 10.1111/dewb.12023 PubMed 23521822 PMC3654560

[33] Geissler PW, Kelly A, Imoukhuede B, Pool R. 'He is now like a brother, I can even give him some blood'—relational ethics and material exchanges in a malaria vaccine 'trial community' in The Gambia. Social science & medicine. 2008;67(5):696–707. Epub 2008/05/06. 10.1016/j.socscimed.2008.02.004 PubMed . 18455854

[34] Edwards SJ. Research participation and the right to withdraw. Bioethics. 2005;19(2):112–30. Epub 2005/06/10. PubMed 15943021. 10.1111/j.1467-8519.2005.00429.x 15943021

[35] Gikonyo C, Bejon P, Marsh V, Molyneux S. Taking social relationships seriously: lessons learned from the informed consent practices of a vaccine trial on the Kenyan Coast. Social science & medicine. 2008;67(5):708–20. Epub 2008/03/26. 10.1016/j.socscimed.2008.02.003 PubMed 18362046 PMC2682177

[36] Scott JA, Bauni E, Moisi JC, Ojal J, Gatakaa H, Nyundo C, et al Profile: The Kilifi Health and Demographic Surveillance System (KHDSS). International journal of epidemiology. 2012;41(3):650–7. Epub 2012/05/01. 10.1093/ije/dys062 PubMed 22544844 PMC3396317

[37] Boga M, Davies A, Kamuya D, Kinyanjui SM, Kivaya E, Kombe F, et al Strengthening the informed consent process in international health research through community engagement: The KEMRI-Wellcome Trust Research Programme Experience. PLoS medicine. 2011;8(9):e1001089 Epub 2011/09/21. 10.1371/journal.pmed.1001089 PubMed 21931539 PMC3172253

[38] Marsh V, Kamuya D, Rowa Y, Gikonyo C, Molyneux S. Beginning community engagement at a busy biomedical research programme: experiences from the KEMRI CGMRC-Wellcome Trust Research Programme, Kilifi, Kenya. Social science & medicine. 2008;67(5):721–33. Epub 2008/04/01. 10.1016/j.socscimed.2008.02.007 PubMed 18375028 PMC2682178

[39] Kamuya DM, Marsh V, Njunguna P, Munywoki P, Parker M, Molyneux S. "When they see us, it's like they have seen the benefits!": experiences of study benefits negotiations in community-based studies on the Kenyan Coast. BMC medical ethics. 2014;15(1):90 10.1186/1472-6939-15-90 PubMed . 25539983 PMC4391117

[40] Green J, Thorogood N. Qualitative Methods for Health Research. Reprint ed. College) DSG, editor: SAGE Publications; 2007. 262 p.

[41] Ayres L, Kavanaugh K, Knafl KA. Within-case and across-case approaches to qualitative data analysis. Qual Health Res. 2003;13(6):871–83. Epub 2003/08/02. PubMed . 12891720 10.1177/1049732303013006008

[42] Ritchie J, Lewis J. Qualitative Research practice: A guide for social science students and researchers Published 2003, reprinted 2009 ed. Ritchie J, Lewis J, editors: SAGE Publications; 2009. 336 p.

[43] Molyneux CS, Murira G, Masha J, Snow RW. Intra-household relations and treatment decision-making for childhood illness: a Kenyan case study. Journal of biosocial science. 2002;34(1):109–31. Epub 2002/01/30. PubMed . 11814209

[44] Lehmann U, Gilson L. Actor interfaces and practices of power in a community health worker programme: a South African study of unintended policy outcomes. Health policy and planning. 2013;28(4):358–66. Epub 2012/07/25. 10.1093/heapol/czs066 PubMed . 22826517

[45] Long N. The Multiple Optic of Interface Analysis (working title). UNESCO Background paper on Interface Analysis1999.

[46] Lukes S. Power A Radical View; 2nd edition second edition ed. Hampshire: Palgrave Macmillian; 2005.

[47] Kandiyoti D. Bargaining with Patriarchy. Gender Soc. 1988;2(3):274–90. PubMed .

[48] Geissler PW. 'Kachinja are coming!': Encounters Around Medical Research Work in Kenyan Vilages. Africa. 2005;75(02):173–202. Epub 03 march 2011.

[49] Molyneux C, Goudge J, Russell S, Chuma J, Gumede T, Gilson L. Conducting health related social science research in low income settings: Ethical dilemmas faced in Kenya and South Africa. Journal of International Development. 2009;21:309–26.

